# Indications, Trends and Outcomes in Pediatric Lung Resections: A 12-Year Study in a Tertiary Referral Center

**DOI:** 10.3390/children12111438

**Published:** 2025-10-23

**Authors:** Gloria Mandrile, Giulia Barone, Vittorio Guerriero, Girolamo Mattioli, Michele Torre

**Affiliations:** 1 Pediatric Surgery Department, IRCCS Istituto Giannina Gaslini, Via Gerolamo Gaslini 5, 16147 Genoa, Italy; giuliabarone1996@gmail.com (G.B.); vittorioguerriero@gaslini.org (V.G.); girolamomattioli@gaslini.org (G.M.); micheletorre@gaslini.org (M.T.); 2 Department of Neuroscience, Rehabilitation, Ophthalmology, Genetics and Maternal and Child Science, University of Genoa, 16132 Genoa, Italy

**Keywords:** pulmonary resections, pediatric thoracic surgery, thoracoscopy

## Abstract

***Background***: Lung resections in children are rare but critical for congenital lung malformations (CLMs) and acquired pathologies; few studies have analyzed the full spectrum of indications. This study evaluated indications, complications, outcomes, and temporal trends in a tertiary pediatric center. ***Methods***: We retrospectively analyzed patients who underwent lung resection (2012–2024), focusing on indications, approaches, complications, and outcomes. Comparisons between pathologies (CLMs vs. acquired pathologies), approaches (thoracoscopy vs. thoracotomy), an temporal trends were evaluated. ***Results***: Among 160 patients (mean age: 7.8 years), acquired lesions (68.6%) were more common than CLMs (31.4%), predominating in children under 8 years. Compared with thoracotomy, thoracoscopy (72.8% of cases, conversion rate: 22.8%) was correlated with shorter operative times (*p* < 0.001) and hospital stays (*p* = 0.001). The complication rate was 19.5%, with 71.9% of patients achieving disease-free, asymptomatic status at follow-up. Risk factors for conversion from thoracoscopy to open surgery included intraoperative adhesions (*p* = 0.003), underlying pathology (*p* = 0.013), and age < 8 years (*p* = 0.017). Compared with acquired lesions, CLMs were associated with fewer complications (14.3% vs. 23.1%, *p* = 0.041) and more favorable outcomes (89.2% vs. 64.7%; *p* < 0.05). Over time, the use of thoracoscopy increased (*p* = 0.012), with reduced operative time (*p* = 0.005); complication and outcome rates remained stable. ***Conclusions***: Pediatric lung resections address diverse pathologies; outcomes are linked to the pathology, and CLMs are associated with lower complication rates in our cohort. Thoracoscopy has progressively become the preferred approach in the last decade, offering advantages particularly in postoperative recovery, though its success depends on careful, pathology-driven patient selection.

## 1. Introduction

Pulmonary resections are infrequently performed in the pediatric population, and the indications for surgery are notably heterogeneous [1,2,3]. Among younger children, particularly those under the age of 8, congenital lung malformations (CLMs) represent the leading indication [1]. These include congenital pulmonary airway malformations (CPAMs), bronchogenic cysts (BCs), bronchopulmonary sequestration (BPS), congenital lobar emphysema (CLE) and congenital bronchial atresia (CBA) [4,5]. In contrast, older children more frequently undergo lung resection for acquired lung conditions, such as complicated pneumonia, suspected or confirmed malignancies, blebs, and small pulmonary nodules requiring biopsy or excision [1,5,6,7].

The literature predominantly focuses on CLMs [8,9,10,11,12,13,14,15], as they are considered among the most pertinent thoracic diseases, with challenging management, which is still a matter of debate [16]; furthermore, lung resections for CLMs can be performed with low morbidity and mortality rates [5]. In contrast, acquired lung lesions frequently have an insidious course, and infective lung diseases, including bronchiectasis and lung abscess, represent the second most important category of lesions in children requiring lung resection after CLMs [2]. However, few studies have comprehensively examined the full spectrum of indications for lung resection in children, mostly based on small cohorts [2,4] or limited data concerning complications and outcomes [1]. However, in our experience, in a tertiary referral center, a broad range of both congenital and acquired lesions are encountered, with patients presenting diverse clinical and anatomical characteristics, for which the overall rates of complications and outcomes have not been precisely described to date.

In recent years, video-assisted thoracoscopic surgery (VATS) has rapidly gained traction as a minimally invasive alternative to open thoracotomy, even for complex lung resections. Numerous studies have demonstrated the safety and feasibility of thoracoscopic approaches, contributing to a paradigm shift in pediatric thoracic surgery [6,17,18]. However, the benefits of thoracoscopic procedures over thoracotomies are still a matter of debate; conflicting evidence persists regarding differences in operative time, postoperative complication rates, and length of hospital stay [8,13,15,19,20]. Despite this, recent reviews suggest that a minimally invasive approach should be employed where possible, even if often it is not the preference of pediatric surgeons for interventions such as lobectomies, as adequate training could be difficult to obtain considering the low incidence of CLMs, the anatomic approach to dissection is different than open surgery, and the size of instruments may be not appropriate for smaller children [20].

In this context, we conducted a retrospective single-center study to explore the heterogeneity of pediatric lung resections in a tertiary referral hospital. We hypothesized that minimally invasive approaches would yield advantages in terms of recovery-related outcomes when compared to open approaches, that CLMs and acquired lesions would show distinct clinical and surgical outcomes, and that significant evolution in surgical practice would be evident over the study period.

## 2. Materials and Methods

We conducted a retrospective single-center study including all patients who underwent pulmonary surgery between January 2012 and December 2024 at the Department of Pediatric Surgery, Giannina Gaslini Institute, a tertiary referral center in Italy. All patients subjected to lung resection procedures were included, whereas those who underwent pleural procedures (such as drainage positioning or pleurodesis) without lung parenchymal resection were excluded from the study. No age restrictions were applied.

We collected data on patient characteristics (sex, medical and surgical history), clinical presentation, indications for surgery, surgical details (approach, procedure performed, operative time), complications (intra- and postoperative, classified according to the Clavien–Dindo classification [21]), length of hospital stay (LOS), and outcomes. Procedures performed were classified in lobectomies, segmentectomies and atypical resections (including all non-anatomical wedge resections). Indications for surgery were categorized into the following pathological subgroups: CLMs (including CPAM, BC, BPS, CLE and CBA), infection-related conditions (pulmonary abscess, bronchiectasis, atelectasis), neoplastic lesions (metastases, primary lung tumors, infiltrating neoplasia), undetermined pulmonary lesions requiring biopsies (including only cases with non-specific histological findings for which a wedge resection was performed, e.g., interstitial lung disease or chronic inflammatory lesions), primary spontaneous pneumothorax (PSP), iatrogenic lesions. A favorable outcome was defined as a disease-free, asymptomatic status at the follow-up evaluated at the most recent outpatient visit or hospitalization for pneumological assessment; patients with evidence of oncologic progression or disease recurrence were categorized as having a non-favorable outcome.

First, we compared differences in operative time, length of stay, complications and outcomes between thoracoscopies and thoracotomies, depending on the type of surgery performed. Patients data were analyzed according to the final surgical approach completed (per-protocol analysis), with procedures converted from thoracoscopy to thoracotomy classified as thoracotomies; this was performed to accurately compare the outcome profiles associated with the successful execution of each technique. A secondary intention-to-treat (ITT) analysis of operative time was also performed, including all patients initially scheduled for thoracoscopy in the thoracoscopy group, regardless of conversion.

Outcomes were analyzed after stratifying patients by pathological diagnosis. Iatrogenic lesions were excluded because of the small sample size (*n* = 2). Subsequently, factors associated with conversion from thoracoscopy to open surgery were evaluated, including pathological subgroups, patient age (categorized as <1 year and <8 years), history of respiratory infections, comorbidities and intraoperative adhesions. The analysis of previous respiratory infection was not applied to the infection-related conditions subgroup to avoid confounding.

Patients were then divided into two groups on the basis of the type of lung lesion: congenital (CLMs) or acquired. The acquired lung lesions included infection-related conditions, neoplastic lesions, lesions requiring biopsies, PSP, and iatrogenic lesions. Differences in surgical indications were assessed in relation to patient age, with 8 years used as the cutoff on the basis of published data [1]. We compared the following parameters between the two groups: symptoms, surgical approach and interventions, complications and outcomes. Reinterventions were excluded from the analysis.

Finally, we assessed temporal trends in indications, surgical approaches, complications and outcomes over the 12-year study period by evaluating potential changes in their frequency and distribution over time.

Data management and statistical analysis were performed with Microsoft Excel (Microsoft Corporation, Redmond, WA, USA), GNU PSPP version 1.6.2 (Free Software Foundation, Boston, MA, USA), and GraphPad Prism version 10.0.0 for Windows (GraphPad Software, Boston, MA, USA). Data normality was assessed via the Shapiro-Wilk test. Qualitative variables are described as percentages and were analyzed via the chi-square test (with Yates correction when appropriate) or Fisher’s exact test. The quantitative variables are presented as the means ± standard deviations and were analyzed via Welch’s *t* test or Student’s *t* test. Trends over time were calculated via simple linear regression for normally distributed data and Spearman’s correlation for nonnormally distributed data. Statistical significance (*p* value) was set as 0.05.

## 3. Results

### 3.1. Population and Procedures

A total of 160 patients who underwent 169 surgical procedures were included in the study group. Patient characteristics are summarized in Table 1.

**Figure 1:**
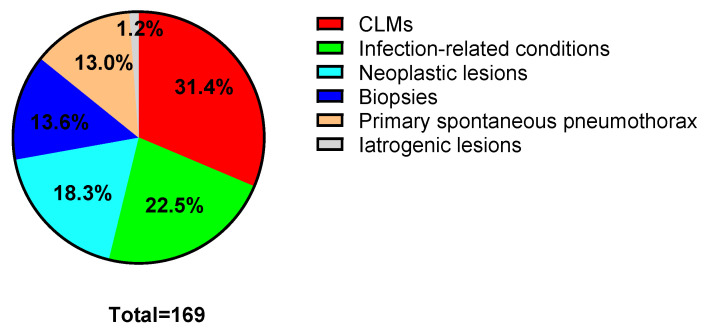
Overall indications for surgery.

The indications for surgery are presented in Figure 1. The most common indication was CLMs (31.4%, 53 patients in total), while 116 patients underwent surgery for acquired lesions. No iatrogenic lesions requiring lung resection were observed after 2015. There were 94 patients less than 8 years of age: 50 (53.2%) had CLMs, whereas 44 (46.8%) had acquired lesions. There were 75 patients over 8 years of age; 3 had CLMs (4.0%), and the other 72 had acquired lesions (96.0%).

We performed 61 lobectomies, 10 segmentectomies and 98 atypical resections (including all non-anatomical wedge resections). The surgical approach was thoracotomy in 46 patients (27.2%) and thoracoscopy in 123 patients (72.8%), of whom 4 were robot assisted (3 for neoplastic lesions, 1 for infection-related conditions). Specifically, a thoracoscopic approach was used in 81% of patients with CLMs (*n* = 43), 55% of those with infection-related conditions (*n* = 21), 48% of patients with neoplastic lesions (*n* = 15), 100% of patients undergoing biopsy (*n* = 23), and 91% of patients with primary spontaneous pneumothorax (*n* = 20). The mean operative time was 129.4 ± 80.9 min. The mean time of thoracoscopy was 92.2 ± 54.2 min, excluding the converted and robotic procedures; the mean time for thoracotomy was 154.1 ± 83.8 min. The overall conversion rate was 22.8% (*n* = 28). The mean duration of hospital stay was 12.8 ± 16.7 days.

Intra- and post-operative complications occurred in 33 patients (19.5%), for a total of 40 adverse events (Table 2). Among the postoperative complications, 3 were managed conservatively (Clavien-Dindo 1), 6 required medical therapy (Clavien-Dindo 2), 11 underwent endoscopic treatment (Clavien-Dindo 3a), 9 underwent pleural drain placement, and 6 underwent surgical reintervention (Clavien-Dindo 3b).

The median follow-up time was 1 year (range: 1 month–9 years); in total, 11 patients were lost to follow-up. The outcome of the first surgical procedure was favorable in 71.9% of patients (*n* = 115); 3.1% of patients (*n* = 5) manifested recurrent infections at follow-up; 8.1% (*n* = 13) had stable disease or oncological progression; death occurred in 6 patients because of underlying pathology. Reintervention for disease recurrence was needed in 6 patients, and 4 patients experienced contralateral recurrence of the disease, with the need for reintervention in 3 patients. One patient needed three surgical procedures.

### 3.2. Thoracoscopy vs. Thoracotomy

The differences between the two surgical approaches are shown in Table 3. A total of 74 thoracotomies were performed during this period, including 28 converted thoracoscopic procedures (10 CLMs, 8 infectious-related conditions, 6 neoplastic lesions, 3 biopsies, 1 iatrogenic lesion). Mean age was significantly higher in the thoracoscopy group. A shorter operative time and LOS were observed for thoracoscopies. Thoracoscopic procedures also resulted in a lower complication rate and higher rate of favorable outcomes, but the difference was not statistically significant.

The analysis stratified by pathological subgroup is shown in Table 4. Mean age was significantly higher in the thoracoscopic groups for patients with PSP and neoplastic lesions. Operative time was significantly shorter for CLMs, infectious conditions, and neoplastic lesions; there was no difference between the two approaches at the ITT analysis. For infectious conditions, thoracoscopy showed reduced LOS and higher favorable outcomes, but higher complication rate. For neoplastic lesions, it was associated with higher complication rates. Minimally invasive approach was predominant for biopsies and PSP, with reduced LOS in the biopsies group.

### 3.3. Conversion to Open

The analysis of factors associated with conversion from thoracoscopy to open surgery (Table 5) revealed significant differences across pathological subgroups, with lower conversion rates found for PSP compared to the other pathologies. Age under 8 years was associated with a significantly higher overall conversion rate, whereas age under 1 year alone did not demonstrate a significant difference. The presence of intraoperative adhesions emerged as a strong predictor of conversion; previous respiratory infections and comorbidities did not significantly influence overall conversion rates.

### 3.4. Congenital Lung Malformations vs. Acquired Lesions

As shown in Table 6, almost all patients with acquired lesions had symptoms for their condition, in contrast to those with CLMs. The rate of successful thoracoscopies was greater for CLMs (61.5% vs. 55.6%), with a higher conversion rate (23.8% vs. 21.1%), but the difference was not statistically significant. Atypical resections were performed more frequently for acquired lesions. Interventions for CLMs demonstrated a shorter LOS, lower complication rates and more favorable outcomes than those performed for acquired lesions. When specific samples of acquired lesions were considered, there was no significant difference in the complication rate (*p* = 0.19) or outcome (*p* = 0.46) across the different conditions. Specifically, the complication rates were 34.2% for infection-related conditions, 31.8% for PSP, 19.3% for neoplastic lesions, and 8.7% after biopsy; the outcome was favorable in 44.7% of patients with infection-related conditions, 68.2% for PSP, 58.1% for neoplastic lesions, and 60.9% for biopsies.

### 3.5. Trends over the Time

**Figure 2:**
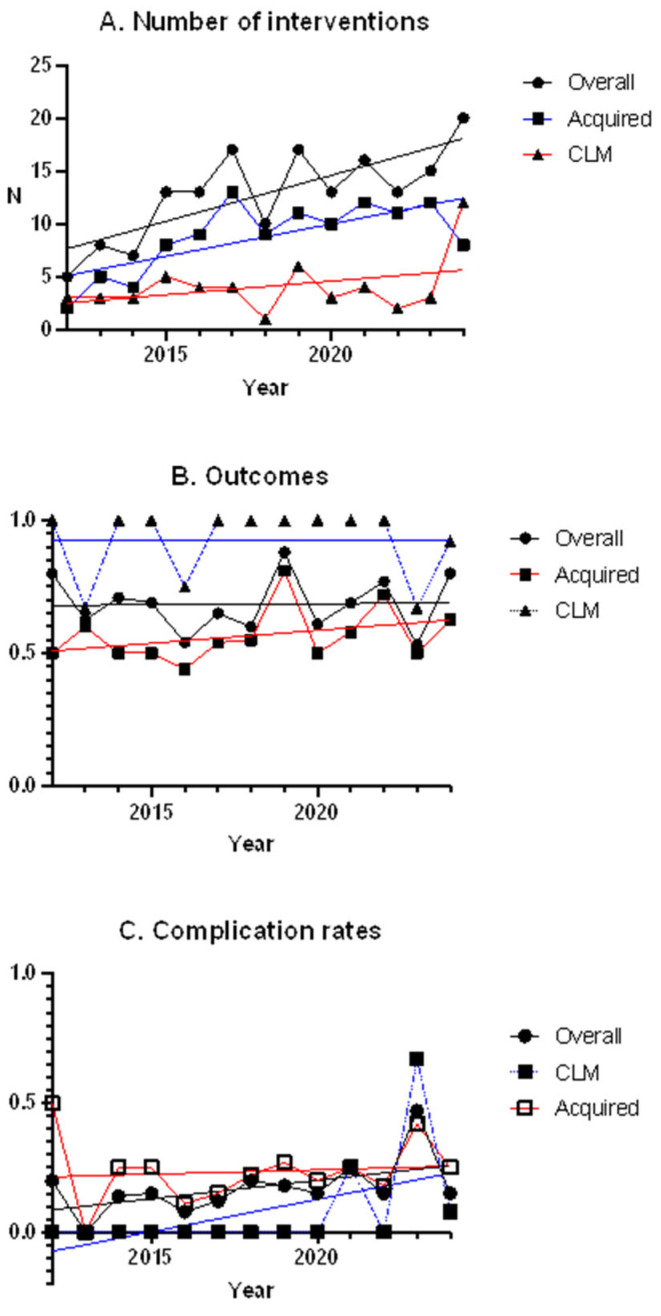
Linear regression: (**A**) Number of procedures performed; (**B**) Outcomes; (**C**) Complication rates (including all intra- and post-operative events). Each parameter calculated for all surgical indications, acquired lesions, and CLMs.

The trends are shown in Figure 2. Over time, we recorded an increase in the number of overall lung surgical procedures performed (r = 0.78, *p* = 0.002), with a stable rate of complications and favorable outcomes. The number of interventions performed for CLMs has remained stable, with an increase in the complication rate. The number of interventions performed for acquired lesions increased (r = 0.71, *p* = 0.007), and the rates of complications and outcomes remained stable

**Figure 3:**
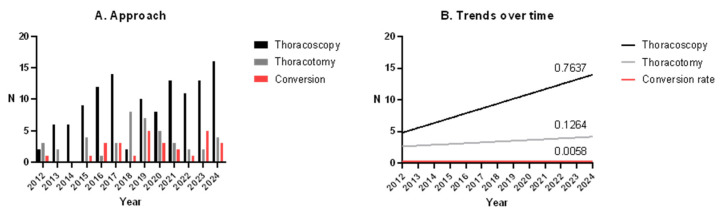
(**A**) Interleaved bars of number of thoracotomies, thoracoscopies and converted thoracoscopies. (**B**) Linear regression for trends over time.

The number of thoracoscopic procedures increased over time (r = 0.67, *p* = 0.012), with a stable conversion rate (Figure 3). With the exclusion of procedures needing conversion, the operative time for thoracoscopic interventions decreased (*p* = 0.005, r = 0.295).

## 4. Discussion

Our 12-year retrospective single-center study highlights the heterogeneity of patients subjected to lung resections in a pediatric tertiary referral hospital. In contrast to most literature focused primarily on CLMs [8,9,10,11,12,13,14,15], the primary aim of this study is to reflect the broad spectrum of conditions encountered in real-world pediatric thoracic surgery. We analyzed outcomes across different pathologies, compared the two main surgical approaches of thoracoscopy and thoracotomy, and evaluated the evolution of practice over the last decade.

In our cohort, acquired lesions constituted the primary indications for surgery, with CLMs representing 31.4% of patients, a lower proportion than reported by Fleming et al. [4] (47.4%), Roy Choudhury et al. [2] (60%), and Clark et al. [22] (73.9%). Notably, these studies focused on cohorts under 18 years of age. This discrepancy suggests that the proportion of acquired lesions may increase with patient age. In support of this hypothesis, in our cohort, acquired lesions were the indication for surgery in 96% of patients older than 8 years, compared with 46.8% among those under this age limit, in line with findings reported by Böckle et al. [1]. All procedures were performed by pediatric surgeons, unlike some institutions where adolescents are managed by adult thoracic units [1]. These observations underscore the importance of considering a broad range of ages and pathologies when evaluating surgical indications in a tertiary referral center. In such setting, pediatric surgeons are often called to manage not only children, but also young adults with complex or long-standing diseases, for which their expertise may be particularly relevant. For this reason, we did not impose an upper age limit on our cohort.

Even though pulmonary resections performed in our center were mostly atypical, the proportion of anatomical resections was greater than that reported by Böckle et al. [1] (42.1% vs. 23%), and we adopted an initial thoracoscopic approach more frequently (72.8% vs. 62%). Our overall complication rate (19.5%) was slightly lower than that reported by Roy Choudhury et al. [2] (22.8%) but higher than that reported by Fleming et al. [4] (10.2%). The management of complications was not clearly described in the cited studies; in our series, most of them required disobstructive bronchoscopies or pleural drain placement, whereas in 6 cases surgical reintervention was necessary.

Few studies in the literature address long-term outcomes following pediatric lung resection for both congenital and acquired conditions. For example, Roy Choudhury et al. [2] reported a mortality rate of 8.6%, higher than that reported in our series (3.7%); Fleming et al. [4] reported the rate of postoperative respiratory infections within one year after surgery (5.3%); however, in the cited studies, the status of patients at the follow-up was not specified. Instead, we provide a comprehensive overview: favorable outcomes, defined as the absence of disease and asymptomatic status at follow-up, were observed in 71.9% of patients; stable disease or oncologic progression occurred in 8.1% of patients, while 3.1% of patients experienced recurrent infections. Reintervention for disease recurrence was needed in six patients (6.7%): four due to infection-related conditions (bronchiectasis and pulmonary abscess), two due to CLMs, and one due to PSP. This represents a strength of our study, which provides a comprehensive overview of pediatric lung resections performed for both congenital and acquired conditions.

### 4.1. Thoracoscopy vs. Thoracotomy

The choice of surgical approach in our series was primarily guided by the type and complexity of the underlying lung pathology, with thoracoscopy employed whenever feasible. Consequently, uncomplicated CLMs, PSP, and diagnostic biopsies were managed thoracoscopically (respectively, 81%, 91% and 100% of cases), while thoracotomy was more commonly reserved for complex congenital or acquired lesion, particularly those of neoplastic origin.

Our analysis revealed unexpectedly significantly shorter operative times for thoracoscopic procedures than for thoracotomies. This finding contrasts with the study of Clark et al., who found no difference [22]. This efficiency is partially attributable to the high proportion of quicker wedge resections performed thoracoscopically. However, even for lobectomies, thoracoscopy remained faster, in contrast with several studies reporting longer [8,17,23,24] or similar times [13,19,20,25]. This result may reflect the standardized and time-efficient nature of this procedure in our center for selected patients. Conversely, the longer operative times in the thoracotomy group likely reflect the inherent complexity of these cases, often requiring conversion from an initial thoracoscopic approach.

Our secondary intention-to-treat analysis of operative time offered a refined perspective. While the reduction in operative time for thoracoscopy remained statistically significant for the entire cohort, it was not significant within individual pathological subgroups. This indicates that the efficiency benefit of thoracoscopy is most consistent when the procedure can be completed without conversion, underscoring the importance of preoperative assessment and patient selection.

In accordance with the literature [15,17,19,22], the LOS after thoracoscopy was shorter than that after thoracotomy but greater than reported in most studies: in our cohort, it was 9.6 ± 13.8 days, whereas in the series of Clark et al. 2 ± 3 days [22], in the one of Polites et al. 2.5–3 days [15], and Kulaylat et al. reported 4.2 ± 5.4 days [19] (the last two studies included only CLMs). This finding is attributed to the heterogeneity and complexity of the patients included in our cohort, some of whom had infective diseases requiring long-term intravenous treatment or systemic pathologies requiring long hospitalizations (tumors, autoimmune diseases, interstitial diseases, and Crohn’s disease), independently of surgical post-operative course.

Consistent with the literature, thoracoscopic resections were also associated with lower complication rates and more favorable outcomes, although the differences did not reach statistical significance. Similar results were reported in the studies of Clark et al. [22], Nasr et al. [13], Dingemann et al. [17], and Polites et al. [15]; in contrast, Kulayat et al. reported fewer complications for thoracoscopic procedures with significant differences [19].

The stratified analyses by pathological subgroup confirmed a consistent reduction in operative time with a thoracoscopic approach across all major pathologies. Furthermore, minimally invasive surgery emerged as the predominant technique for biopsies and PSP. For infectious conditions, thoracoscopy offered a shorter LOS and higher rate of favorable outcomes, but increased complication rates. For neoplastic lesions, the higher complication rate was not counterbalanced by superior recovery metrics. This indicates that the impact of the surgical technique and the resulting risk-benefit profile are not uniform across different disease processes. However, the interpretation of these results is inherently limited by the significant imbalance in patient distribution between the two surgical approaches within each pathological subgroup. These findings underscore that the comparative performance of thoracoscopy and thoracotomy is highly dependent on the underlying pathology. Furthermore, the imbalanced distribution itself is a clinically meaningful result, indicative of real-world surgical decision-making process and evidence the critical importance of careful patient selection in complex subgroups.

The risk of conversion from thoracoscopy to open surgery was primarily determined by the underlying pathology and the presence of intraoperative adhesions, consistent with findings from Clark et al. [22]; this underscores the importance of preoperative assessment for minimally invasive surgery. Contrary to previous reports [22,26], a history of respiratory infections did not significantly predict conversion in our cohort; this may be partially explained by the proactive surgical approach to CLMs before infections occur. Neoplastic and infectious pathologies presented the highest conversion rates, likely reflecting the technical challenges inherent in these conditions. The complete absence of conversions in PSP reinforces the well-documented suitability of thoracoscopy for this subgroup [1,17]. While age under 8 years was associated with increased conversion rates, the lack of significance in infants under 1 year supports the feasibility of thoracoscopy in youngest patients [3] and suggests that pathological factors rather than chronological age alone drive conversion risk, consistent with Clark et al. [22]. Notably, aside from adhesions in the neoplastic subgroup, no other factors reached statistical significance in the subgroup analyses, likely due to limited sample sizes, whereas the analysis of the overall cohort provided sufficient power to identify these key risk factors.

These results across a broad spectrum of pediatric pulmonary diseases confirm the general advantages of thoracoscopy, while delineating its specific benefits and limitations in different clinical scenarios and emphasize the necessity for individualized patient selection based on specific pathological characteristics.

### 4.2. Congenital Lung Malformations vs. Acquired Lesions

Our study highlighted substantial differences between CLMs and acquired lung lesions (Table 6). In contrast to the literature, the rate of thoracoscopic procedures was higher in CLMs, although this difference was not statistically significant; in contrast, Kulaylat et al. [19] reported a predominance of open resections for CLMs.

Atypical resections were significantly more common in acquired conditions, likely reflecting the surgical approach to treating diseases such as PSP and diagnostic lung biopsies. The complication rate for CLMs in our series (7.7%) was notably lower than that previously reported: in the meta-analysis of Liu et al., it was 17.9% [8]; in the series of Kulaylat et al., it was 18.6% [19]; Musters et al. reported 11% in thoracoscopic procedures only [18]; and Farolfi et al. reported acute complications in 31.2% of cases and 73.7% long-term sequelae, including frequent respiratory symptoms [10], whereas favorable outcomes were reported in 94.2% of cases in our series at follow-up. In contrast, interventions for acquired lesions were associated with a higher complication rate (22%) and a lower rate of favorable outcomes (61.1%). These results are slightly different from those reported in previous studies: Ayed et al. [27] and Fan et al. [28] reported lower morbidity rates (16% and 13.5%, respectively) after lung resection for infectious pulmonary disease [27]. Sırmalı et al. [29] reported 73.3% favorable outcomes, with a morbidity rate of 13% [29], for the same type of intervention. Nevertheless, comparison remains challenging, as most studies focus on specific pathologies, whereas our study provides a comprehensive overview of surgical outcomes across diverse indications to reflect the real-world practice in pediatric thoracic surgery in a tertiary referral center.

### 4.3. Trends over Time

As shown in Figure 2, our analysis revealed a significant increase in the number of overall lung surgical procedures performed from January 2012 to December 2024, particularly for acquired lesions. The number of interventions performed for CLMs slightly increased, without reaching statistical significance. This contrasts to the reported increase in the incidence of these malformations [26] and the extended indication to perform elective surgery in asymptomatic patients with CLMs [30], which was adopted in our center. Our result may be partially explained by the rarity of these pathologies, as the number of affected patients remains relatively small. However, in the last year included in the study, patients operated for CLMs were 12 (versus 3 in the first years of the study period); this sharp increase suggests a likely continued upward trend in the coming years. We also observed an increase in the complication rate for CLMs over the study period. While this may reflect a true increase in adverse events, it is also plausible that this trend is influenced, at least in part, by enhanced detection and reporting mechanisms. These include the implementation of more meticulous postoperative documentation, the adoption of standardized monthly complication reports, and improved data capture through digital systems at our institution.

The number of thoracoscopic procedures performed at our institution has increased significantly over time (Figure 3). This result is consistent with those of previous studies, which confirmed that VATS has become a safe and feasible approach, even for complex lung resections, and is now widely accepted as a minimally invasive alternative to open thoracotomy [3,6,17,18]. Similarly, increased use of thoracoscopy has been reported in pediatric series both for all lung resections [22] and specifically for CLMs [15,16].

The overall conversion rate from thoracoscopy reported in our study was 22.8%, remaining stable throughout the study period; the operative time decreased, indicating a significant learning curve. These findings are comparable to the reported conversion rates in the literature, ranging from 1.4% to 33% [6]. Clark et al. reported a conversion rate of 22% [22], whereas Lieber et al. reported a conversion rate of 13.2%, with a significant learning curve during the study period [6].

Taken together, these temporal trends highlight the dynamic and evolving landscape of pediatric thoracic surgery, reflecting technical advancements and shifts in clinical judgment.

### 4.4. Limitations of This Study

Despite the valuable insights gained from this study, several limitations must be acknowledged. The retrospective design carries inherent risks of information bias, as reliance on existing medical records may lead to incomplete or inaccurate data. No advanced statistical methods were employed to reduce bias. While standardized inclusion criteria and centralized electronic data collection provided some consistency, key factors such as the surgeon’s experience or learning curve, which could influence outcomes and complications were not measured or adjusted for in the analysis.

Significant selection bias influenced the choice of surgical approach. The decision to perform thoracoscopy or thoracotomy was not randomized but based on surgeon assessment and lesion characteristics, with more complex cases likely directed toward open surgery; this explains the imbalance in patient distribution between the two surgical approaches in the pathological subgroups. While this limits direct comparability, it reflects real-world surgical decision-making.

Furthermore, the core aim of describing the heterogeneity in pediatric lung resections at a tertiary referral center represents both a strength and a fundamental limitation. Although our cohort size is substantial compared to many retrospective pediatric surgical series [2,4,6,22,31], the analyzed groups were profoundly heterogeneous. This encompasses a wide age range (neonates to young adults), diverse pathologies (congenital vs. acquired lesions), and varied surgical procedures (minor resections to complex lobectomies). This variability limits our ability to establish standardized benchmarks for complication rates or outcomes across different patient subgroups or procedure types.

Finally, as all patients were treated in a tertiary referral center, the cohort likely enriched with complex cases and severe pathologies requiring specialized care. Consequently, the observed outcomes may not be generalizable to the broader pediatric population undergoing lung surgery in non-tertiary contexts.

## 5. Conclusions

Pulmonary resections in children encompass a highly heterogeneous group of patients, pathologies, and surgical procedures. This large 12-year single-center experience indicates that outcomes are largely influenced by the underlying disease, with congenital lesions generally associated with fewer complications and more favorable results compared to acquired conditions. Our analysis identified intraoperative adhesions, specific pathological subtypes (particularly neoplastic and infectious conditions), and younger age (<8 years) as significant risk factors for conversion from thoracoscopy to open surgery. Over time, thoracoscopic surgery progressively became the preferred approach, offering advantages particularly in postoperative recovery. Our findings support the continued adoption of minimally invasive techniques and emphasize the importance of pathology-driven, individualized surgical planning in pediatric lung resections.

## Figures and Tables

**Table 1:** Patient characteristics.

		*n* (%)
**Sex**	Male	82 (51.2%)
	Female	78 (48.8%)
**Age**	Mean	7.8 years
	Standard deviation	6.3 years
	Range	3 days–24.4 years
**Clinical presentation**	Symptomatic cases	121 (71.6%)
	Respiratory infections	60 (35.5%)
	Pneumothorax	22 (13.0%)
	Respiratory distress/dyspnea	13 (7.7%)
	Thoracic pain	7 (4.1%)
	Cough	6 (3.5%)
	Systemic symptoms	6 (3.5%)
	Others (Pleural effusion, hemoptysis, hemothorax)	7 (4.1%)
	Asymptomatic	48 (28.4%)

**Table 2:** Complications of lung resection procedures.

		*n* (%)
**Intraoperative**	Major intra-thoracic injuries	4 (2.3%)
	Bleeding	2 (1.2%)
**Short-term postoperative**	Pneumothorax	10 (5.9%)
	Atelectasis	8 (4.8%)
	Wound infections	4 (2.4%)
	Hematoma/bleeding	2 (1.2%)
	Subglottic stenosis	2 (1.2%)
	Nerve lesions	2 (1.2%)
	Others (pneumonia, pleural effusion, thoracic drain dislocation)	3 (1.8%)
**Long-term postoperative**	Bronchial obstruction	1 (0.6%)
	Broncho-pleural fistula	1 (0.6%)
	Pneumothorax	1 (0.6%)

**Table 3:** Comparison between thoracoscopy and thoracotomy.

	Thoracoscopy	Thoracotomy	*p* Value
**Total**	95	74	
Age, month (mean ± st.dev.)	107.2 ± 81.2	75.3 ± 63.9	0.005 *
Males, *n* (%)	53 (55.8%)	34 (45.9%)	0.218
**Operative time,** min (mean +/− st.dev.)			
Overall	93.2 ± 54.0	175.2 ± 85.9	<0.001 *
Lobectomies	132.7 ± 66.9	200.6 ± 93.2	0.002 *
Segmentectomies	105.8 ± 77.9	145.0 ± 49.5	0.542
Atypical resections	79.1 ± 37.5	146.3 ± 67.5	<0.001 *
**Operative time ITT,** min (mean ± st.dev.)			
Overall	120.2 ± 78.2	154.1 ± 84.7	0.022 *
Lobectomies	175.8 ± 93.0	178.2 ± 91.6	0.919
Segmentectomies	115.7 ± 82.1	135.0 ± 65.6	0.710
Atypical resections	95.7 ± 55.6	121.9 ± 67.4	0.149
**Length of stay**, days (mean ± st.dev.)			
Overall	9.6 ± 13.8	17.9 ± 19.8	0.001 *
Lobectomies	8.5 ± 11.1	20.6 ± 23.5	0.010 *
Segmentectomies	4.5 ± 2.1	8.5 ± 5.0	0.262
Atypical resections	10.3 ± 15.0	15.7 ± 14.4	0.104
**Complications**, *n* (%)			
Overall	17 (17.9%)	16 (21.6%)	0.681
Lobectomies	6 (28.6%)	10 (25%)	0.763
Segmentectomies	1 (16.7%)	0 (0%)	0.389
Atypical resections	10 (14.7%)	6 (20%)	0.513
**Favorable outcome**, *n* (%)			
Overall	69 (72.6%)	49 (66.2%)	0.464
Lobectomies	16 (76.2%)	27 (67.5%)	0.479
Segmentectomies	4 (66.7%)	2 (50.0%)	0.598
Atypical resections	53 (77.9%)	20 (66.7%)	0.239

* Statistical significance (*p* value < 0.05). ITT = Intention To Treat. Complications included all intra- and post-operative events.

**Table 4:** Comparison between thoracoscopy and thoracotomy by pathological subgroup.

Pathological Subgroup	Variable	Thoracoscopy	Thoracotomy	*p* Value
**CLMs**		**33 (62.2%)**	**20 (37.7%)**	
	Age, months	27.6 ± 31.9	29.3 ± 49.3	0.890
	Males	19 (57.6%)	6 (30%)	0.088
	Operative time, min	113.1 ± 62.6	172.2 ± 90.1	0.010 *
	Operative time ITT, min	141.8 ± 83.0	108.0 ± 52.2	0.119
	LOS, days	6.4 ± 9,4	11.1 ± 10.0	0.105
	Complications	3 (9.1%)	2 (11.1%)	0.902
	Favorable outcome	31 (93.9%)	19 (95.0%)	0.819
**Infection-related conditions**		**13 (34.2%)**	**25 (65.8%)**	
	Age, months	111.5 ± 45.3	100.8 ± 58.8	0.542
	Males	7 (53.8%)	12 (48.0%)	1
	Operative time, min	113.2 ± 60.3	188.9 ± 81.7	0.003 *
	Operative time ITT, min	141.8 ± 79.7	189.3 ± 80.8	0.078
	LOS, days	10.6 ± 16.6	26.3 ± 19.6	0.015 *
	Complications	8 (61.5%)	5 (20.0%)	0.028 *
	Favorable outcome	11 (84.6%)	7 (28.0%)	0.002 *
**Neoplastic lesions**		**9 (29.0%)**	**22 (71.0%)**	
	Age, months	155.1 ± 79.2	91.0 ± 51.7	0.047 *
	Males	2 (22.2%)	12 (54.5%)	0.132
	Operative time, min	92.5 ± 45.4	170.1 ± 96.5	0.007 *
	Operative time ITT, min	139.1 ± 88.2	157.7 ± 97.0	0.594
	LOS, days	13.0 ± 23.0	11.9 ± 8.3	0.892
	Complications	4 (44.4%)	2 (9.1%)	0.043 *
	Favorable outcome	5 (55.5%)	15 (68.2%)	0.683
**Biopsies**		**20 (86.9%)**	**3 (13.1%)**	
	Age, month	133.8 ± 76.2	83.3 ± 82.7	0.404
	Males	9 (45.0%)	2 (66.7%)	0.590
	Operative time, min	58.1 ± 32.7	183.3 ± 62.9	0.069
	Operative time ITT, min	74.4 ± 56.1	None	
	LOS, days	13.3 ± 16.8	5.0 ± 1.0	0.041 *
	Complications	2 (10.0%)	0 (0.0%)	1
	Favorable outcome	12 (60.0%)	2 (66.7%)	0.759
**PSP**		**20 (90.9%)**	**2 (9.1%)**	
	Age, month	187.5 ± 38.7	1.5 ± 0.7	<0.001 *
	Males	16 (80.0%)	1 (50%)	0.411
	Operative time, min	81.1 ± 33.1	92.5 ± 3.5	0.179
	Operative time ITT, min	Unchanged	Unchanged	
	LOS, days	8.7 ± 9.3	14.5 ± 3.5	0.179
	Complications	5 (25.0%)	0 (0.0%)	1
	Favorable outcome	14 (70.0%)	2 (100%)	0.190

* Statistical significance (*p* value < 0.05). Qualitative variables: *n* (%); quantitative variables: mean +/− st.dev. CLMs = Congenital Lung Malformations. ITT = intention to treat. LOS = Length Of hospital Stay. PSP = primary spontaneous pneumothorax; note: there was no conversion from thoracoscopy to thoracotomy, so the operative time ITT does not change from the per-protocol analysis. Complications included all intra- and post-operative events.

**Table 5:** Analysis of factors associated with conversion from thoracoscopy to open.

Associated Factors	Variable	Attempted Thoracoscopy	Converted to Open	*p* Value
**Pathological subgroup**	Total	122	27 (22.1%)	0.013 *
	CLMs	43	10 (23.3%)	0.823
	Infection-related conditions	21	8 (38.1%)	0.080
	Neoplastic lesions	15	6 (40.0%)	0.097
	Biopsies	23	3 (13.0%)	0.402
	PSP	20	0 (0.0%)	0.006 *
**Age < 1 year**	Total	27	6 (22.2%)	1
	CLMs	25	5 (25.0%)	0.717
	Infection-related conditions	1	1 (100%)	0.381
	Neoplastic lesions	0		N/A
	Biopsies	1	0 (0.0%)	1
	PSP	0		N/A
**Age < 8 years**	Total	65	20 (30.8%)	0.017 *
	CLMs	40	9 (22.5%)	0.558
	Infection-related conditions	10	5 (50%)	0.387
	Neoplastic lesions	6	4 (66.7%)	0.136
	Biopsies	8	2 (25.0%)	0.269
	PSP	0		N/A
**Previous respiratory infections**	Total	27	4 (14.8%)	0.774
	CLMs	15	1 (6.7%)	0.127
	Neoplastic lesions	4	3 (75.0%)	0.235
	Biopsies	6	0 (0.0%)	0.539
	PSP	2	0 (0.0%)	1
**Comorbidities**	Total	58	13 (22.4%)	0.942
	CLMs	4	1 (25.0%)	1
	Infection-related conditions	18	7 (38.9%)	1
	Neoplastic lesions	10	2 (20%)	0.089
	Biopsies	17	3 (17.6%)	0.539
	PSP	9	0 (0.0%)	1
**Adhesions**	Total	14	8 (57.1%)	0.003 *
	CLMs	3	2 (66.7%)	0.130
	Infection-related conditions	2	2 (100%)	0.133
	Neoplastic lesions	3	3 (100%)	0.044 *
	Biopsies	4	1 (25.0%)	0.453
	PSP	2	0 (0.0%)	1

* Statistical significance (*p* value < 0.05). Qualitative variables: *n* (%). CLMs = Congenital Lung Malformations. PSP = primary spontaneous pneumothorax; note: when no attempted thoracoscopy was performed, *p*-value was not calculated.

**Table 6:** Comparison between CLMs and acquired lesions.

	CLMs	Acquired Lesions	*p* Value
**Number**	52	108	
**Symptomatic patients**, *n* (%)	23 (44.2%)	99 (91.7%)	<0.001 *
**Operative time**, min (mean ± st.dev.)	135.4 ± 78.1	126.2 ± 81.9	0.486
**Length of stay**, days (mean ± st.dev.)	8.2 ± 9.8	15.5 ± 19.3	0.001 *
**Type of interventions**, *n* (%)			
Successful thoracoscopies	32 (61.5%)	60 (55.6%)	0.473
Converted thoracoscopies	10 (23.8%)	17 (21.1%)	0.478
Thoracotomies	10 (19.2%)	32 (42.1%)	0.161
Lobectomies	25 (48.1%)	35 (32.4%)	0.081
Segmentectomies	4 (7.7%)	6 (5.6%)	0.861
Atypical resections	23 (44.2%)	75 (69.4%)	0.004 *
**Complications,** *n* (%)	4 (7.7%)	24 (22.2%)	0.041 *
**Favorable Outcome,** *n* (%)	49 (94.2%)	66 (61.1%)	<0.001 *

* Statistical significance (*p* value < 0.05). CLMs = Congenital Lung Malformations. Complications included all intra- and post-operative events.

## Data Availability

The original contributions presented in this study are included in the article. Further inquiries can be directed to the corresponding author.
